# On the Role of Stimulus-Response Context in Inhibitory Control in Alcohol Use Disorder

**DOI:** 10.3390/jcm11216557

**Published:** 2022-11-04

**Authors:** Filippo Ghin, Christian Beste, Ann-Kathrin Stock

**Affiliations:** 1 Cognitive Neurophysiology, Department of Child and Adolescent Psychiatry, Faculty of Medicine, TU Dresden, Fetscherstrasse 74, 01309 Dresden, Germany; 2 University Neuropsychology Center, Faculty of Medicine, TU Dresden, Fetscherstrasse 74, 01309 Dresden, Germany; 3 Biopsychology, Faculty of Psychology, School of Science, TU Dresden, Zellescher Weg 10, 01069 Dresden, Germany

**Keywords:** alcohol use disorder, cognitive control, EEG, response inhibition

## Abstract

The behavioral and neural dynamics of response inhibition deficits in alcohol use disorder (AUD) are still largely unclear, despite them possibly being key to the mechanistic understanding of the disorder. Our study investigated the effect of automatic vs. controlled processing during response inhibition in participants with mild-to-moderate AUD and matched healthy controls. For this, a Simon Nogo task was combined with EEG signal decomposition, multivariate pattern analysis (MVPA), and source localization methods. The final sample comprised n = 59 (32♂) AUD participants and n = 64 (28♂) control participants. Compared with the control group, AUD participants showed overall better response inhibition performance. Furthermore, the AUD group was less influenced by the modulatory effect of automatic vs. controlled processes during response inhibition (i.e., had a smaller Simon Nogo effect). The neurophysiological data revealed that the reduced Simon Nogo effect in the AUD group was associated with reduced activation differences between congruent and incongruent Nogo trials in the inferior and middle frontal gyrus. Notably, the drinking frequency (but not the number of AUD criteria we had used to distinguish groups) predicted the extent of the Simon Nogo effect. We suggest that the counterintuitive advantage of participants with mild-to-moderate AUD over those in the control group could be explained by the allostatic model of drinking effects.

## 1. Introduction

Alcohol Use Disorder (AUD) is a chronic disease characterized by uncontrolled drinking, despite negative consequences [1], and is one of the leading causes of morbidity and mortality in European countries [2]. Most current therapeutic approaches primarily are aimed at reducing craving and/or attenuating the reinforcing effects of alcohol [3,4,5]. Still, patients often fail to remain abstinent for longer periods after the treatment programs end [6,7]. For these reasons, there is growing interest in investigating potential alternative treatment approaches [8,9]. Ideally, those approaches should be derived from a better understanding of the functional cognitive and neurobiological mechanisms underlying the development and maintenance of AUD [10].

It has repeatedly been suggested that hazardous consumption in AUD patients is mainly driven by excessively strong alcohol-related habits and automatic stimulus-response associations [6,8]. Additionally, a progressive impairment of cognitive control functions further hinders appropriate response selection and inhibition in AUD [11,12]. Inhibitory control functions are essential in conflicting contexts where, for example, an automatic stimulus-response association may not be appropriate. The execution of automatic behavior itself is not very taxing on cognitive resources, but considerable resources are required when such automatic behavior must be inhibited. In contrast, a more controlled response requires a higher initial allocation of cognitive resources to be correctly executed, but little inhibitory control is required when it must be suppressed [13,14]. In this regard, it has been suggested that controlled processing, which is required to adhere to explicit goals or task instructions, depends on effortful top-down volitional processes. In contrast, automatic processes, which are triggered by certain stimulus features and do not always serve the pursuit of explicit goals, operate effortlessly and partly on stimulus-response associations [14,15,16]. In the following, we will use this distinction as a working definition for automatic and controlled processes investigated in the current study.

Yet still, cognitive control and automaticity should not be considered as separate entities, but as interacting factors that share overlapping neural networks [17,18,19,20]. In healthy individuals, these somewhat opposing modes of generating behavior are in relative balance. However, it has been suggested that this balance may be disrupted in AUD patients [9,10]. Nevertheless, it remains unclear whether and how the imbalance between automated and controlled processes during inhibitory control is linked to drinking behavior and relapse rates in AUD. The current study systematically investigates this question while also considering the neurophysiological level.

To this end, we used a relatively recently developed experimental paradigm combining the Simon task and the Go/Nogo task, which allows the examination of the interplay of controlled and automated processing during goal-directed actions [21,22,23]. For the Simon task, the dual-route model [24] states that conflicts arise due to competing automatic and controlled (response) selection processes. The controlled processes are induced by the identity of the target stimulus (in our case, whether an “A” or a “B” is shown), which is the only stimulus feature important for correct responding by using either the left or right index finger. However, the task-irrelevant spatial position of the target stimulus (left or right on the screen), triggers an unconditional (automatic) selection of the response located on that same side. This automatic response tendency has to be controlled (inhibited) when the spatial position of the stimulus requires a response on the opposing side/finger (incongruent trials). This interplay of the so-called direct (automatic) route and the indirect (controlled) route is essential to explain behavior: In congruent trials, the direct route is sufficient for correct responding, but, in incongruent trials, the indirect route also needs to be activated to ‘control’ the direct route. When processing via the direct route dominates, response inhibition becomes harder and more error-prone, ultimately resulting in more inhibition failures in congruent trials compared with incongruent trials, where the indirect (controlled) route already exerts some control over the direct (automatic) route [21,23,25]. As a consequence of these mechanisms, the “regular” Simon effect is characterized by better behavioral performance in congruent than in incongruent Go trials. In contrast to this, the Simon Nogo effect is inverted and characterized by worse behavioral performance in congruent than in incongruent Nogo trials [25,26]. Based on this, the Simon Nogo paradigm can help the understanding of how the level of automaticity in a stimulus-response context modulates both response selection and inhibition processing in AUD [22] and how this is linked to drinking behavior and relapse rate.

To examine associated neurophysiological processes, we pursued two complementary strategies. First, we examined traditional event-related potential (ERPs) components, which have repeatedly been shown to reflect distinct subprocesses involved in inhibitory control [27]. Because the N2 and P3 reflect distinguishable response inhibition subprocesses [27], we focused on the N2-P3 ERP complex in the current study. Additionally, we used a data-driven approach employing multivariate pattern analysis (MVPA) of our EEG data [28,29,30,31]. MVPA can be used to decode information representing differences between experimental conditions (classes) based on segmented EEG data [32,33,34,35]. As opposed to more traditional approaches, MVPA allows the capture of the full information contained in the EEG signal (i.e., its multivariate nature) [36], and thus the identification of differences that may otherwise be missed when confining analyses to classic ERPs. This is especially advantageous in conditions where neurophysiological processes are likely to change in broad/spatially distributed neural networks.

Importantly, different aspects of information are concomitantly coded in the EEG signal [36,37]. This has been shown to be particularly evident when investigating the interplay of automatic and controlled processes during response selection and inhibition [21,38]. For this reason, we further applied a residue iteration decomposition (RIDE) [39,40], which can dissociate between stimulus-related processing codes and stimulus-response mapping (response selection) codes in separate clusters of activity in the EEG signal (i.e., the S-cluster and the C-cluster, respectively). It was previously shown that information gained from both clusters is relevant for understanding how the level of automaticity in a stimulus-response context modulates response selection and inhibition [21]. Therefore, the ERP and MVPA analyses of the current study were conducted on RIDE-decomposed EEG data to investigate how automatic vs. more controlled processing modulates response inhibition performance in mild-to-moderate AUD and healthy controls. Lastly, we performed source localization analyses to examine which functional neuroanatomical structures are associated with the most distinct neural activity differences between conditions and groups. This information may be particularly crucial to further develop and refine non-invasive brain stimulation approaches targeted to treat AUD [10].

## 2. Materials and Methods

### 2.1. Participants and Recruitment Procedure

Participant eligibility was assessed via a telephone interview. We only included participants in the age range from 18 to 40 years. The interview included a brief version of the SCID structured clinical interview of the DSM-5 and questions regarding the applicants’ medical history. Applicants were not included in the study if they reported having developed any withdrawal symptoms that might impair wellbeing or performance during a sober test appointment. Further exclusion criteria were prior medical, neurological, and mental illness records except for AUD, tobacco use disorder, and acute or lifetime cannabis addiction. Eligible participants had no acute or chronic medication intake or other recreational drug addiction that could affect the central nervous system on the day of testing. After the recruitment, eligible participants were invited to participate in the study. For each included AUD participant, a healthy control participant matched by age (±2 years) and sex was recruited. At the beginning of the experimental session, participants were asked to read and sign the informed consent form. Next, breath alcohol concentration (BrAC) was assessed using the breathalyzer “Alcotest 3000” (Drägerwerk, Lübeck, Germany). Participants were required to be sober (BrAC = 0.00%) and were also asked to provide a urine sample for drug testing (SureStep^TM^, Innovacon Inc., San Diego, CA, USA). Positive results on substances except for cannabis metabolites (i.e., THC-COOH) also resulted in the termination of the study appointment. The complete list of tested drugs and metabolites is provided in the Supplementary Materials. Participants received EUR 50 as monetary compensation. All applied procedures were approved by the Ethics Committee of the medical faculty of the Technical University of Dresden (EK 513122018) and conducted in accordance with the Declaration of Helsinki.

### 2.2. Clinical Assessment

After the initial BrAC and drug assessment, participants were asked to fill in a series of questionnaires and participate in a structured interview designed to assess their alcohol consumption, depression symptoms, and mental cognitive abilities (additional sociodemographic information and questionnaires [ASSIST and BIS-15] were also collected but not analyzed nor considered relevant for the current study, so they are not reported in detail). Specifically, alcohol abuse and dependence were assessed using the complete version of the structured clinical interview (SCID) for the DSM-5 [41]. The SCID-5 for AUD is designed for diagnosing AUD according to the DSM-5 criteria for substance use disorder. The SCID provides information about the past 12 months and lifetime AUD diagnosis based on 11 criteria. The number of fulfilled criteria then determines the severity of the disorder. Severity levels are ranked as mild (2–3 criteria), moderate (4–5 criteria), and severe AUD (≥6 criteria). A past year diagnosis of AUD is confirmed when a minimum of 2 criteria are fulfilled for the past 12 months. On the other hand, a lifetime AUD diagnosis requires at least two fulfilled criteria preceding the previous 12 months [7]. The amount of past-year AUD criteria determined the allocation of the participants to the AUD group (≥2 AUD criteria) or the control group (≤1 AUD criteria). Thus, only the past year’s AUD diagnosis was used to allocate participants to their respective groups. Other alcohol consumption indices were also evaluated. The Alcohol Use Disorders Identification Test (AUDIT) [42] was administered to screen for hazardous and harmful alcohol consumption. Furthermore, participants were asked about their daily drinking in the past three months to assess drinking frequency and binge-drinking behavior. Specifically, participants were asked, “*On how many days did you drink at least one glass of alcohol in the last three months?*” (i.e., drinking frequency) and “*On how many days within the last three months did you drink five or more glasses of alcohol?*” (i.e., binge drinking). Finally, participants were also asked to complete the Mini-Mental State Examination (MMSE) and the Beck Depression Inventory (BDI).

### 2.3. Behavioral Task

**Figure 1:**
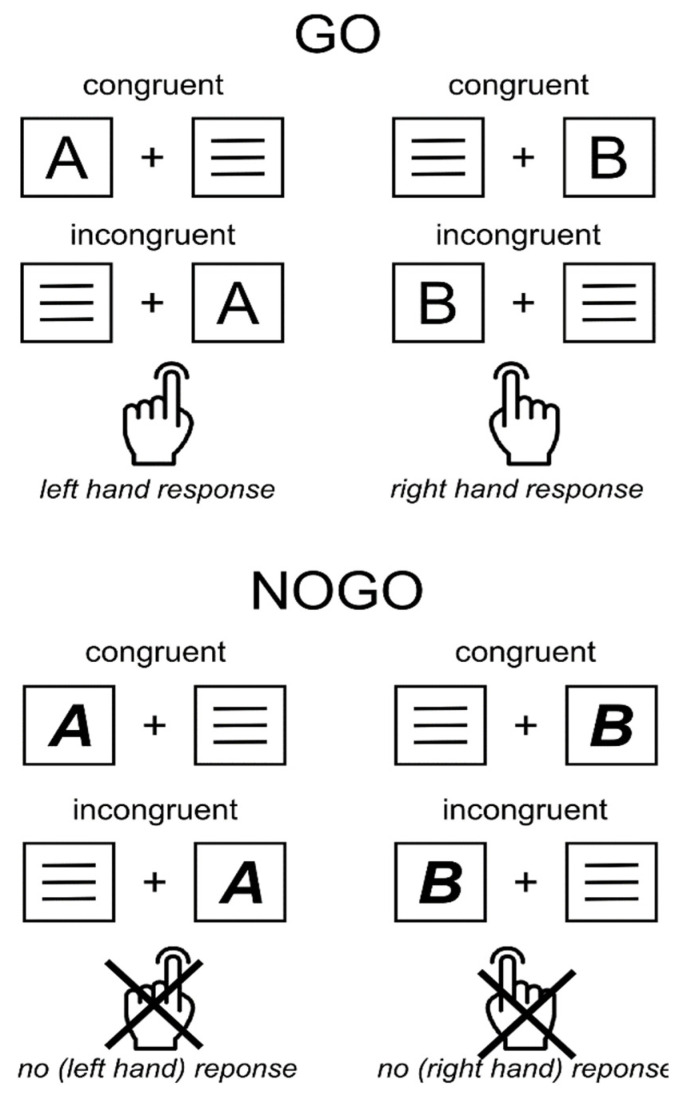
Experimental Simon Nogo paradigm. All possible stimulus-response combinations are illustrated for the Go condition (**top panel**) and the Nogo condition (**bottom panel**).

To measure the effect of automatic vs. controlled processes on response inhibition, an integration of Simon and Go/Nogo paradigms was used (Figure 1) [22,23,43].

Participants were comfortably seated in front of a 24” CRT monitor at a distance of 57 cm. A white central fixation cross was continuously presented in the center of the screen and sided by two lateralized white frame boxes against a black background. This visual setup was constantly displayed for the entire duration of the task. On each trial, a single yellow letter stimulus in a standard “Arial” font (i.e., letter “A” or “B”) was displayed for 200 ms within either the left or the right white frame box. At the same time, a distractor stimulus made of three horizontal white lines was presented within the opposing box. At the beginning of the task, participants were instructed to place their left and right index fingers on the left and right Ctrl buttons of a standard German “QWERTZ” keyboard and maintain the position of their fingers throughout the entire task duration. When the letter stimuli were presented in standard font, participants were instructed to respond to each standard letter stimulus as fast as possible by pressing the left or right control button (Go trials). Participants were asked to press the left Ctrl button whenever the letter “A” was presented and press the right Ctrl button whenever the letter “B” was presented, regardless of the spatial position of the letter within the frame boxes. Importantly, whenever the letters were displayed in a combined ***bold***–***italic*** font (i.e., “***A***”/“***B***”), participants were instructed to inhibit any response (Nogo trials). Trials in which the letter stimulus was presented in the spatial location of the corresponding response hand were coded as congruent trials (i.e., “A” presented on the left, “B” presented on the right). Trials in which the stimulus letter was presented in the opposite spatial location were coded as incongruent (i.e., “A” presented on the right, “B” presented on the left). Following this logic, the experimental paradigm consisted of four primary conditions: (1) congruent Go trials; (2) incongruent Go trials; (3) congruent Nogo trials; and (4) incongruent Nogo trials. Participants performed a total of 720 trials, 70% (504 trials) of which were Go trials and 30% (216 trials) of which were Nogo trials. For both Go and Nogo trials, each target letter was presented equally often and both target letters were presented with the same frequency in congruent (50%) and incongruent (50%) stimulus-response mappings. The inter-trial interval (ITI) was jittered between 1300 and 1700 ms. Go trials were coded as “hits” when a correct response was registered within a time window of 0 to 1700 ms after stimulus onset. Incorrect responses within a time window of 0 to 1700 ms were coded as “errors”. When no response was registered within 1700 ms, these Go trials were coded as “misses”. Nogo trials were coded as correct omissions when no response was registered within 1700 ms after stimulus onset. Any response obtained in Nogo trials between 0 and 1700 ms after stimulus onset was coded as a “false alarm”. The ITI started when a response was registered, or when 1700 ms had elapsed without any response. The experimental paradigm was divided into six blocks of 120 trials each. In each of the blocks, the frequency of trial types and conditions was kept equal. After each block, participants could take a self-timed break and continue the task with a button press. The entire experiment took ~30 min to complete. Presentation (Neurobehavioral Systems Inc., Berkeley, CA, USA) was used to program the experiment and to record behavioral data. After the Simon Nogo task, two other, but unrelated, experimental paradigms were run.

### 2.4. EEG Recording and Analysis

The EEG signal was acquired from 60 equidistant Ag-AgCl-electrodes using a QuickAmp amplifier (Brain Products GmbH, Gilching, Germany). The reference electrode was positioned at Fpz (θ = 90, φ = 90) and the ground electrode was positioned at the coordinates θ = 58, φ = 78. During recording, the sampling rate was 500 Hz. Offline, the data were pre-processed using the software Brain Visual Analyzer (Brain Products GmbH). Specifically, data were down-sampled to 256 Hz, and a band-pass filter (IIR) between 0.5 and 40 Hz employing a slope of 48 dB/oct each, and a 50 Hz notch filter was applied. Channels presenting a noisy signal or a flat line during recording were removed, and recordings were re-referenced to the average activity of all remaining electrodes. Raw data were then inspected manually to remove technical non-periodic artifacts. Periodically occurring artifacts such as eye blinks, saccades, and cardiovascular artifacts were corrected using an independent component analysis (ICA; Infomax algorithm). Any remaining artifacts were removed during a second manual inspection of the data. Finally, a topographic interpolation was applied to reconstruct previously discarded channels. Importantly, only data from trials with correct behavioral responses (i.e., Go trials with hits and Nogo trials with correct omissions) were included in the analysis. The data were separately segmented for the four trial conditions. Each segment started at −2000 ms and ended at 2000 ms with respect to the stimulus onset. An automatic artifact rejection procedure was applied to remove segments with a maximum value difference of more than 200 μV in a 200 ms time window and/or a minimum of less than 0.5 μV in a 100 ms time window. Afterwards, a current source density (CSD) transformation was applied (order of splines = 4, maximal degree of Legendre polynomials = 10, and approximation parameter Lambda = 0.0005). This transformation provided a reference-free evaluation of the electrophysiological data [44]. Finally, a baseline correction was applied within the time interval from −300 ms to 0 ms before the target stimulus onset.

#### 2.4.1. Residue Iteration Decomposition (RIDE)

RIDE was applied to the pre-processed and segmented single-subject EEG data [40]. This approach has been adopted successfully in previous studies [29,45,46,47]. RIDE applies an iteration scheme decomposing single-trial data while retaining channel-specific information to assess the identified components. Because the RIDE algorithm is blind to scalp distributions and waveforms, the application of a CSD does not affect the results (Ouyang et al., 2015). In short, the RIDE method decomposes EEG data into different temporal components representing distinct, but partially overlapping, sensory and cognitive processes. Temporal components can be extracted by static latency information of specific events like stimulus onset (S-cluster) and response marker (R-Cluster). The S-cluster is thought to represent information associated with perception and attentional processes, while the R-cluster is thought to represent response processes that are associated with motor preparation and motor execution processes. A third cluster is related to central processes (i.e., C-cluster) between the S- and R-clusters and is associated with stimulus evaluation and response selection processes. Because temporal components within the C-cluster are not locked to time markers, their latency information is estimated and iteratively improved. The current study employed the RIDE toolbox (https://cns.hkbu.edu.hk/RIDE_files/Page308.htm accessed on 13 October 2022) implemented in Matlab (Mathworks Inc., Natick, MA, USA). Notably, the R-Cluster cannot reliably be determined in Go/Nogo tasks since motor execution rarely occurs in Nogo trials. Thus, the current decomposition only included the S-cluster and the C-cluster. RIDE clusters were derived using a time window function. That is, each time window is thought to include specific components. For this study, a time window from −200 before until 400 ms after the stimulus onset was implemented for the S-Cluster. For the C-cluster, a time window starting from 200 until 800 ms after stimulus onset was implemented. Of note, this is in line with previous publications using the same task [21]. Once the time-markers are provided, RIDE decomposes EEG data using an iterative method applying an L1-norm minimization (i.e., obtaining median waveform) to improve the components’ convergence. A comprehensive overview of the RIDE method and the iterative procedure can be found elsewhere [39,40,48,49].

For an analysis of event-related potentials (ERPs) after applying RIDE, the following electrodes were selected based on a visual inspection of the ERPs and their scalp topography: For the S-cluster, the N2 component was quantified as the mean activity at electrode Cz from 275 to 295 ms and at FCz from 295 to 315 ms. The P2 component was quantified at electrodes FCz and Cz from 180 to 200 ms. For the C-cluster, the P3 ERP was separately quantified for fronto-central and centro-parietal electrodes. At fronto-central electrodes, the mean activity was quantified at electrodes FCz and Cz from 425 to 455 ms for Go trials and from 475 to 505 ms for Nogo trials. At centro-parietal electrodes, the mean activity was quantified from 410 to 440 ms for Go trials and from 440 to 470 ms for Nogo trials at electrode Pz, and from 420 to 450 ms for Go trials and from 470 and 500 ms for Nogo trials at electrode CPz. For the statistical analyses using the mean amplitudes in the mentioned time windows, the data were pooled across the chosen electrodes for the quantification of each ERP component. For the analyses, we used mixed-effects ANOVAs with condition (Go vs. Nogo) and congruency (congruent vs. incongruent) as within-subject factors and group (AUD vs. control) as between-subject factors.

#### 2.4.2. Multivariate Pattern Analysis (MVPA)

The MVPA was applied to the RIDE-decomposed EEG data using the MVPA light toolbox [35]. A binary classification across time was used to identify the time window(s) showing significant difference patterns between congruent and incongruent Nogo trials. Only signals within a time window of 0–1500 ms from the stimulus onset were used for MVPA. A two-class L1-Support Vector Machine (SVM) classifier was chosen to contrast congruent and incongruent Nogo trials because of its recognized robustness in dealing with outliers. Specifically, the SVM method has shown better performance when applied to either noisy or non-Gaussian-distributed data [35]. For the binary classification computation, a five-fold cross-validation method was applied. At the same time, all other parameters were set to the standard setting of the MVPA light toolbox (i.e., the dataset is randomly split into five folds). In each iteration, one fold was used for testing, while the remaining four folds were used for training. This procedure was then repeated until every fold had once been used as the test set. Importantly, no repetition was used for cross-validation. The area under the ROC curve (AUC) was used to evaluate classification accuracy. The AUC is a non-parametric effect-size measure derived from signal detection theory that can be used to assess classifier performance [50]. AUC refers to the total area considered when plotting cumulative true positive rates against false positive rates. A cluster-based permutation analysis with 1000 random draws based on the non-parametric Wilcoxon tests (*p* = 0.05) was used to detect the time points with a significant classification performance represented by the area under the curve (AUC). The sum of all Wilcoxon-test values within the investigated time range was employed for the cluster level statistic. The null value for AUC was a chance level of 0.5 (i.e., 50%).

#### 2.4.3. Source Localization

To locate functional neuroanatomical structures associated with the RIDE-decomposed ERPs and MVPA-detected time windows, the source location method sLORETA (standardized low-resolution brain electromagnetic tomography; Pascual-Marqui, 2002) was used. sLORETA uses a realistic MNI152 head model and divides the intracerebral volume into 6239 voxels using a spatial resolution of 5 mm [51]. A standardized current density for each voxel is then calculated [52]. sLORETA is a source localization method providing a linear solution to the inverse problem without localization bias [53,54]. sLORETA was used to compare neural activity patterns between congruent and incongruent Nogo trials in the S- and C-clusters. To do this, the sLORETA built-in voxel-wise randomization test with 2000 permutations was used based on statistical non-parametric mapping (SnPM) to correct for multiple comparisons. Voxels located in the MNI brain template showing a significant difference (*p* < 0.05) are shown in the Section 3.

## 3. Results

### 3.1. Sample Characteristics

*N* = 148 participants were invited to the study. *N* = 15 participants were excluded from the analyses for the following reasons. Two participants were used to pilot the technical setup and subsequently excluded as we corrected a few minor settings after collecting their data. Three participants were excluded because they reported moderate symptoms of depression (BDI score > 19). Three were excluded due to positive results in the urine drug-screening test. Data from two participants could not be used due to technical issues with the experimental equipment. One participant was excluded due to a prior history of meningitis not reported in the initial telephone screening. For two participants, it was impossible to determine the correct number of AUD criteria during the clinical assessment due to conflicting information provided by the participants. Finally, two participants were excluded because behavioral performance in the task was below the chance level (most likely caused by an erroneous understanding of the task instructions). Behavioral (i.e., accuracy) data were then separately assessed in each task condition and group for possible outliers by using the Turkey method approach implemented in SPSS. This resulted in the identification of ten outliers. Therefore, analyses were conducted on a final sample of n = 123 participants, n = 59 (32 males) of which were in the AUD group, and n = 64 (28 males) of which were in the control group. Sociodemographic and alcohol consumption indices for the two groups are presented in Table 1 and Table 2, respectively. Detailed frequency data of 1-year and lifetime AUD criteria for AUD and control groups are reported in Table S2 in the supplementary material. Notably, the AUD and the control groups did not differ in age and mental state (MMSE score), but showed significant differences in years of education and the amount of reported depressive symptoms (BDI, see Table 1). However, it is worth noting that BDI scores in both groups indicated only minimal depression symptoms.

### 3.2. Behavioral Results

**Figure 2:**
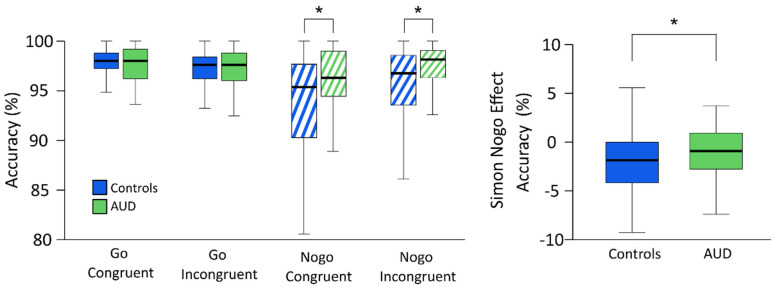
Behavioral results. The box plots illustrate the mean accuracy in percent (%) for the four experimental conditions (**left**) and Simon Nogo task effect (**right**). Accuracy in the Go condition was measured in correct responses, whereas accuracy in the Nogo condition was a measure of correct omissions. The Simon Nogo effect was calculated as a difference between congruent and incongruent Nogo trials. Asterisks (*) indicate significant differences at *p* < 0.05. Error bars represent 95% confidence intervals.

Results for response accuracy in the Simon Nogo task are illustrated in Figure 2.

Repeated-measures ANOVA results for main and interaction effects are reported in Table 3.

Results for the main effects showed higher accuracy in the AUD group (97.06% ± 0.35) than in the control group (95.7% ± 0.33), higher accuracy in Go trials (97.33% ± 0.17) than in Nogo trials (95.44% ± 0.40), and higher accuracy in incongruent (96.61% ± 0.24) than in congruent trials (96.16% ± 0.26). Bonferroni-corrected post hoc comparisons investigating the task-typical significant interaction of condition × congruency showed higher overall accuracy in congruent trials (97.65% ± 0.16) than in incongruent trials (97.01% ± 0.21) in the Go condition (*t* = 3.629, *p* = 0.002). On the other hand, a significantly higher overall accuracy in incongruent trials (96.15% ± 0.38) than in congruent trials (94.60% ± 0.48) was found in the Nogo condition (*t* = −6.087, *p* = 0.002). These results were also confirmed by additional Wilcoxon signed-rank tests for Go (*z* = −3.049, *p* = 0.004) and Nogo trials (*z* = −5.234, *p* = 0.002) and align with previous findings using the same experimental paradigm on healthy participants [21,23].

To investigate the significant three-way interaction of group × condition × congruency, additional post hoc repeated measures ANOVAs with congruency (congruent vs incongruent) as the within-subject factor and group (AUD vs control group) as the between-subject factor were separately run for Go and Nogo conditions. Results showed that, for the Go condition, there was a significant main effect of congruency (*F*_(1,121)_ = 12.866, *p* < 0.001, *η*^2^*_p_* = 0.096; congruent = 97.64% ± 0.16; incongruent = 97.02% ± 0.21), but no significant main effect of group (*F*_(1,121)_ = 0.052, *p* = 0.820, *η*^2^*_p_* < 0.001) or interaction of congruency × group (F_(1,121)_ = 0.794, *p* = 0.375, *η*^2^*_p_* = 0.007). For the Nogo condition, results showed a significant main effect of congruency (*F*_(1,121)_ = 37.016, *p* < 0.001, *η*^2^*_p_* = 0.234; incongruent = 96.20% ± 0.37; congruent = 94.67% ± 0.46), a significant main effect of group (*F*_(1,121)_ = 12.323, *p* < 0.001, *η*^2^*_p_* = 0.092; AUD group = 96.84% ± 0.58; control group = 94.03% ± 0.55), and a significant interaction of congruency × group (*F*_(1,121)_ = 4.628, *p* = 0.033, *η*^2^*_p_* = 0.037). Bonferroni-corrected post hoc comparisons showed that, for both congruent and incongruent Nogo trials, there was a significant difference between the AUD group (congruent = 96.34% ± 0.41; incongruent = 97.33% ± 0.36) and the control group (congruent = 93.00% ± 0.80; incongruent = 95.07% ± 0.63) (parametric tests: all t ≥ |3.109|, all *p* ≤ 0.004; non-parametric tests: all z ≥ |2.647|, all *p* ≤ 0.016). Most importantly, however, group comparisons for the Simon Nogo effect (i.e., congruent minus incongruent trials) showed that it was significantly larger in the control group (−2.07% ± 0.38) than in the AUD group (0.99% ± 0.32) (parametric: *t* = −2.151, *p* = 0.033, Cohen’s d = −0.39; non-parametric: z= −2.001, *p* = 0.045).

#### 3.2.1. Alcohol-Related Modulators of Response Inhibition

Counterintuitively, the results showed that AUD participants had an overall better task performance in terms of accuracy and a smaller effect of the interaction between automaticity and control on response inhibition. To understand these results better, we further investigated whether measures of hazardous alcohol consumption (i.e., current AUD criteria and AUDIT scores), drinking frequency, and binge drinking frequency modulated response inhibition processes in automatic vs. more controlled processes. To do this, separate two-way mixed-effect ANCOVAs were run, each using one alcohol consumption index as a single covariate (i.e., AUD 1-year criteria, or AUDIT, or drinking frequency, or binge drinking frequency). Condition (Go vs. Nogo) and congruency (congruent vs. incongruent) were used as within-subject factors and group (AUD vs. control group) was used as the between-subjects factor. The results of these analyses are reported in Table 4.

Overall, the ANCOVAs showed that group differences in automatic vs. controlled processes in response inhibition were modulated by all alcohol-related consumption indices (i.e., AUD 1-year criteria, AUDIT, drinking frequency, and binge drinking frequency), as evidenced by the changes in the interaction of group × condition × congruency in the respective ANCOVAs. This is particularly interesting considering that response inhibition performance (i.e., the Simon Nogo effect) was less modulated by the response context (automatic vs. controlled processes) in the AUD group than in the control group. Interestingly, the ANCOVA analyses also revealed a significant main effect of drinking frequency, but not of the other alcohol consumption indices. To investigate the relationship between alcohol consumption indices and response inhibition embedded in automatic vs. controlled processes further, a multiple linear regression was conducted across the entire sample, with the behavioral Simon Nogo effect as the dependent variable and all of the aforementioned alcohol consumption indices as predictors. The results showed that only drinking frequency significantly predicted the size of the Simon Nogo effect, while the other factors (i.e., AUD 1-year criteria, AUDIT, and binge drinking), did not. The results of the multiple regression analysis are shown in Table 5.

**Figure 3:**
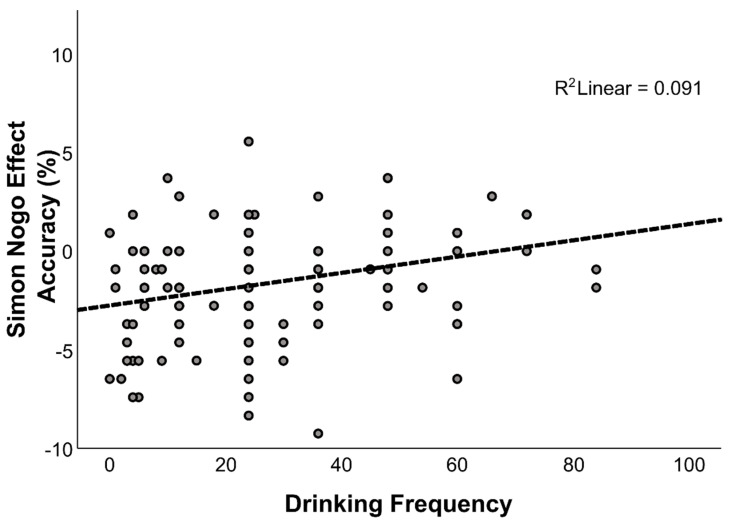
Scatterplot showing the relationship between the Simon Nogo effect (calculated as the difference between congruent and incongruent Nogo trials) and drinking frequency.

Overall, these results showed a significant association between drinking frequency and the Simon Nogo effect. In particular, the results showed that a smaller Simon Nogo effect was associated with a higher drinking frequency, thus providing an explanation of why we found the Simon Nogo effect to be smaller in AUD participants (Figure 3).

#### 3.2.2. Summary of Behavioral Results

Counterintuitively and surprisingly, behavioral results showed that the AUD group had a better response inhibition performance than the control group. While the typical task effects (i.e., the regular Simon effect in Go trials and the inverted Simon effect in Nogo trials) were evident in the entire sample, the AUD group exhibited a smaller Simon Nogo effect than the control group. In other words, AUD participants were characterized by a smaller modulation of response inhibition performance by the level of automaticity vs. control exerted for the prepotent Go response. Furthermore, analyses of covariance showed that alcohol-related indices modulated the relationship between automatic vs. controlled processes in response inhibition. In particular, drinking frequency, rather than the initially devised distinction based on the number of AUD criteria, predicted the size of the Simon Nogo effect, with higher drinking frequency corresponding to a lower modulation of response inhibition performance. Additional add-on analyses investigating the effects of BDI and years of education can be found in the supplementary materials (Tables S3 and S4). Overall, their results showed that BDI did not modulate the pattern of behavioral results, but years of education, similarly to alcohol indices, modulated behavioral performance. Yet, both BDI and years of education could not predict the size of the Simon Nogo effect (all *p* > 0.05).

### 3.3. Neurophysiological Results

#### 3.3.1. S-Cluster

The ANOVA for the N2 amplitudes showed that there were no significant main effects (all *F* ≤ 3.817, all *p* ≥ 0.053), or significant interactions (all *F* ≤ 2.501, all *p* > 0.116).

For the P2 amplitudes, there was a significant main effect of condition (*F*_(1,121)_ = 4.065, *p* = 0.046, *η*^2^*_p_* = 0.033), with lower amplitudes in Go (1.96 µV/m^2^ ±0.18) than in Nogo trials (2.06 µV/m^2^ ± 0.18). Furthermore, there was a significant main effect of group (*F*_(1,121)_ = 4.321, *p* = 0.040, *η*^2^*_p_* = 0.034), with higher amplitudes in the AUD group (2.38 µV/m^2^ ± 0.26) than in the control group (1.64 µV/m^2^ ± 0.25). However, no significant main effect of congruency (*F*_(1,121)_ = 1.406, *p* = 0.238) or any significant interactions (all *F* ≤ 3.490, all *p* > 0.064) were found. Illustrating graphs for the S-cluster are provided in the Supplementary Materials.

#### 3.3.2. C-Cluster

The ANOVA for the fronto-central P3 amplitudes revealed a significant main effect of condition (*F*_(1,121)_ = 293.029, *p* < 0.001, *η*^2^*_p_* = 0.708), with larger amplitudes in Nogo (4.46 µV/m^2^ ± 0.22) than in Go trials (1.31 µV/m^2^ ± 0.20). There was also a significant main effect of congruency (*F*_(1,121)_ = 10.304, *p* = 0.002, *η*^2^*_p_* = 0.078), with larger amplitudes in incongruent trials (3.01 µV/m^2^ ± 0.20) than in congruent trials (2.76 µV/m^2^ ±0.19). No significant main effect of group was found (*F*_(1,121)_ = 1.430, *p* = 0.234, *η*^2^*_p_* = 0.012). The results showed a significant interaction of condition × congruence (*F*_(1,121)_ = 21.607, *p* < 0.001, *η*^2^*_p_* = 0.152). Bonferroni-corrected post hoc comparisons showed larger amplitudes in incongruent Go trials (1.61 µV/m^2^ ± 0.21) than in congruent Go trials (1.02 µV/m^2^ ± 0.20) (*t* = −6.408, *p* = 0.002). No significant difference was found between congruent and incongruent Nogo trials (*t* = 0.662, *p* = 1.000). Furthermore, no other significant interactions were found (all *F* ≤ 2.417, all *p* ≥ 0.123).

**Figure 4:**
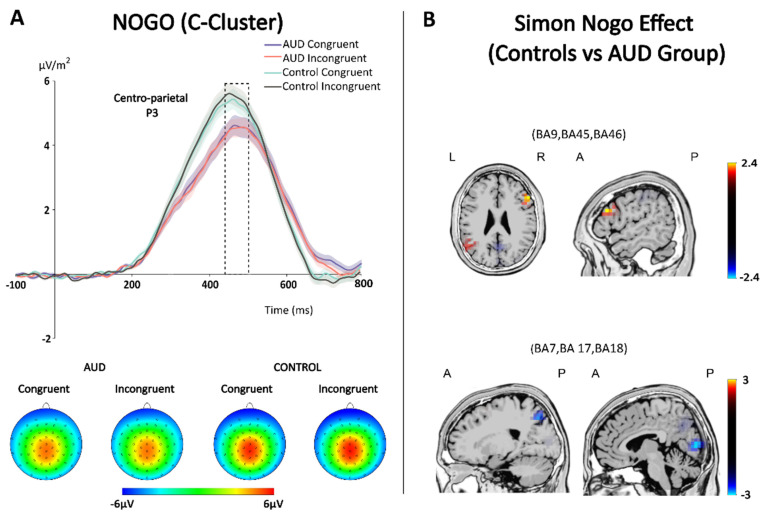
(**A**) The centro-parietal P3 ERP (pooled across Cz and CPz) and corresponding scalp topography maps for Nogo trials in the C-cluster. The different colors of the ERP lines indicate the different experimental groups and conditions. The shaded areas represent the 95% confidence interval estimates. The dashed box illustrates the time window used for the statistical analyses. (**B**) sLORETA-derived maps for the Simon Nogo effect indicate the sources of maximal differences between the control group and AUD group in the dashed time window. Positive values indicate larger activation differences in the control group, and negative values indicate larger activation differences in the AUD group.

The ANOVA for the centro-parietal P3 amplitudes showed a significant main effect of condition (*F*_(1,121)_ = 11.414, *p* < 0.001, *η*^2^*_p_* = 0.086), with larger amplitudes in Go trials (5.32 µV/m^2^ ± 0.25) than in Nogo trials (4.80 µV/m^2^ ±0.22). Furthermore, there was a significant main effect of group (*F*_(1,121)_ = 5.597, *p* = 0.020, *η*^2^*_p_* = 0.044), showing larger amplitudes in the control group (5.57 µV/m^2^ ±0.30) than in the AUD group (4.54 µV/m^2^ ± 0.32). However, no significant main effect of congruency (*F*_(1,121)_ = 0.017, *p* = 0.896) was found. No significant interaction was found for condition × group, congruency × group, and condition × congruency (all *F* ≤ 2.736, all *p* ≥ 0.101). Interestingly, a significant interaction of condition × congruency × group (*F*_(1,121)_ = 4.398, *p* = 0.038, *η*^2^*_p_* = 0.035) was found. To investigate this 3-way interaction, post hoc repeated measures ANOVAs were run separately for Go and Nogo trials using congruency (congruent vs. incongruent) as within-subject factor and group (AUD vs. control) as between-subject factor. For Go trials, results showed a significant main effect of group (*F*_(1,121)_ = 6.251, *p* = 0.014, *η*^2^*_p_* = 0.049; control group = 5.94 µV/m^2^ ± 0.34; AUD group = 4.69 µV/m^2^ ±0.36), but no significant main effect of congruency (*F*_(1,121)_ = 1.167, *p* = 0.282), or significant interaction of congruency × group (*F*_(1,121)_ = 1.011, *p* = 0.317). For Nogo trials, results showed no significant main effects of congruency and group (all *F* ≤ 3.752, all *p* ≥ 0.055). However, a significant interaction of congruency × group was found (*F*_(1,121)_ = 4.461, *p* = 0.037, *η*^2^*_p_* = 0.036). Bonferroni-corrected post hoc comparisons showed no significant group difference in congruent Nogo trials (*t* = 1.545, *p* = 0.25). However, the control group showed larger P3 amplitudes (5.33 µV/m^2^ ± 0.30) than the AUD group (4.35 µV/m^2^ ± 0.30) in incongruent Nogo trials (*t* = 2.298, *p* = 0.046). Additionally, the Simon Nogo effect for the P3 (i.e., congruent minus incongruent P3 amplitudes) was compared across groups (Figure 4). Independent *t*-tests showed a significantly (*t* = −2.112, *p* = 0.037) larger P3 Simon Nogo effect in the control group (−0.24 µV/m^2^ ± 0.10) than in the AUD group (0.05 µV/m^2^ ± 0.10). Source localization analyses showed a larger neural activation difference for the control group than the AUD group in the right inferior frontal gyrus (BA45 and BA9), and the middle frontal gyrus (BA46). In contrast, the AUD group showed a larger neural activation difference in the cuneus (BA17 and BA18) and precuneus (BA7) than the control group. Illustrating graphs for the C-cluster are provided in the Supplementary Materials.

#### 3.3.3. Multivariate Pattern Analysis (MVPA)

**Figure 5:**
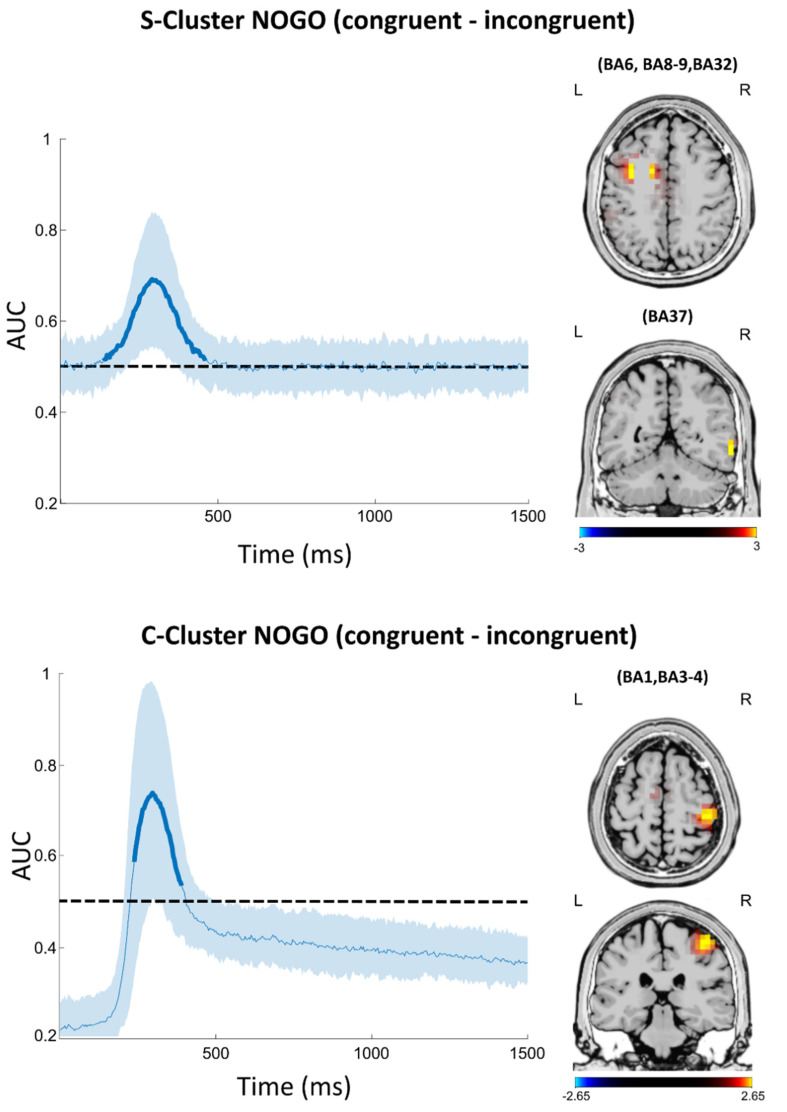
MVPA classification results show the area under the curve (AUC) decoding accuracy across time for all subjects for RIDE data in the S-Cluster (**top left**) and C-Cluster (**bottom left**). Time point zero denotes the onset of target stimulus presentation. Thick lines indicate the significant time windows (*p* < 0.05; two-sided cluster-based permutation). The dashed line indicates the chance level. Shaded error bars represent the standard deviation. Additional sLORETA-derived maps indicate the sources of maximal differences between congruent and incongruent Nogo trials in the highlighted time windows showing above-chance level classification performance for S-Cluster (**top right**) and C cluster (**bottom right**). Positive values indicate larger activation in congruent Nogo trials, while negative values indicate larger activation for congruent Nogo trials.

Against the background that drinking frequency (and not the number of AUD criteria) was most closely associated with variations in the Simon Nogo effect, we decided against comparing groups with this method and to apply multivariate analyses on the entire sample (i.e., combining EEG data from both AUD and control groups) instead. Specifically, MVPA was run to identify time windows in which stimulus-associated processes (S-cluster) and central processes (C-cluster) significantly differed between congruent and incongruent Nogo trials. The results of the MVPA and the sLORETA source localization analysis based on the MVPA findings are illustrated in Figure 5.

In the S-Cluster, the classification was above chance level between 137 ms and 464 ms after stimulus onset, with the AUC reaching a peak of 0.69 at 293 ms after stimulus onset. For this time window, the source localization analysis revealed higher neural activation in congruent than in incongruent Nogo trials for the middle frontal gyrus (BA8 and BA6), precentral gyrus (BA9), cingulate gyrus (BA32) and middle and inferior temporal gyrus (BA37). For the C-cluster, the classification was above chance level between 238 ms and 390 ms after stimulus onset, peaking at 0.74 at 297 ms after stimulus onset. For this time window, the source localization analysis revealed higher neural activation in congruent than in incongruent Nogo trials for the post-central gyrus (BA1 and BA3) and primary motor cortex (BA4).

## 4. Discussion

In the current study, we investigated whether the balance between automated and controlled processes during response inhibition is altered in AUD and how this is linked to drinking behavior. In this context, we also examined the underlying functional neuroanatomical and neurophysiological changes to gain a better understanding of the mechanisms driving AUD-related behavioral changes.

Counterintuitively, our behavioral results showed that AUD participants were characterized by better response inhibition performance and a smaller modulation of this faculty by the level of automaticity vs. control exerted for the prepotent Go response. We further found that alcohol-related indices of drinking modulated the relationship between automatic vs. controlled processes in response inhibition. Specifically, it was not the severity of AUD (reflected by the number of AUD criteria that we used to define groups of AUD patients and control participants), but rather higher drinking frequency, which was associated with a lower modulation of response inhibition performance between congruent and incongruent trials.

In both congruency conditions, AUD participants showed better inhibition performance than healthy control participants. This finding was quite surprising since current evidence indicates that inhibitory control impairments are a major contributing factor in dysregulated drinking and higher relapse rates in AUD [11,12]. However, it should be noted that it is still a matter of debate whether inhibitory control impairments already arise at the early stages of the disorder or can only be found in more severe cases of AUD [55]. It has been suggested that the lack of consistency among studies investigating inhibitory control in AUD could not only be associated with variability in drinking levels among samples, but also with differences in experimental designs [10]. In line with this, the role of stimulus salience during inhibitory control could be another critical factor which may help to explain our results. For example, there is evidence showing that individuals with AUD can produce an inhibition performance comparable to that of controls during a Go/Nogo task when low salience stimuli are used (i.e., circles) [56]. However, inhibition control deficits become evident in AUD when participants are required to respond to alcohol-related stimuli, which are supposedly characterized by a higher salience for AUD, but not necessarily control participants [57,58,59]. Against the background that our study only included participants with mild-to-moderate AUD who were not experiencing withdrawal symptoms, it could hence be possible that either this subgroup has not yet shown response inhibition impairments, or that those impairments are not (yet) generalized and limited to drinking-related behavior only.

On the neurophysiological level, the AUD group showed a larger P2 amplitude in the S-cluster, indicating a better allocation of processing resources in the AUD group than in healthy controls [26,60,61,62]. Importantly, the S-cluster has been associated with stimulus-related processing linked to perception and attention [39,48]. In line with this, there is recent evidence suggesting that when participants are instructed to attend to multiple irrelevant and relevant features of a stimulus, the additional processes necessary for producing the correct response may decrease the automaticity of the prepotent response [63]. If that is the case, the improved response inhibition and reduced Simon Nogo effect in AUD may be due to a stronger increase in the attentional processing resources required to resolve multiple conflicting aspects of target stimulus information. This should in turn cause a greater reduction of automatic processing during response selection and thereby facilitate response inhibition.

Yet, such a generalized change in attentional processing is insufficient to explain the finding that AUD seems to be associated with a reduced distinction between direct and indirect route processing in Nogo trials at both the behavioral and the neurophysiological level. There are several studies that provided interesting insight into the dynamics of context-related modulations on response selection and response inhibition. In this context, an alternative explanation for our findings could be provided by allostatic processes associated with alcohol consumption and by the differential mechanisms underlying cognitive processing in congruent vs. incongruent trials.

As detailed in the introduction, Simon task effects can be explained by the dual-route model [24], which postulates competing automatic and controlled response selection processes in incongruent trials. As previously shown by our group, this interplay is also relevant in response inhibition [21,25,26]. Given that neither the general performance nor the performance in incongruent trials seemed to be worse in the AUD group, it can be deduced that increased drinking (at least to the degree investigated in our sample) does not seem to substantially damage processing via the indirect route at the investigated AUD stage. The decreased Simon Nogo effect further suggests that processes mediated via the direct and the indirect route may become less differentiated with increasing frequency of alcohol consumption. In terms of the dual-route model, such a de-differentiation performance can be possible if there is an overall decrease in direct route processing and/or an overall increase in indirect route processing (i.e., in both congruent and incongruent trials), but our data do not allow us to conclude to which degree each of these changes took place. The allostatic model of drinking proposes that repeated drinking episodes induce adaptive changes in various neurotransmitter systems. In short, the concept of allostasis refers to the fact that repeated or chronic changes to a given (neuro)transmitter system are counter-balanced by (initially) adaptive changes away from the normal homeostatic range, so that normal functioning can only be sustained in the presence of those causal factors [64,65,66,67,68]. In case of frequently repeated alcohol consumption, this mechanism leads to the downregulation of the GABAergic system and the upregulation of the glutamatergic system [68]. While the allostatic model itself only refers to changes in basic neurotransmitter processes, it is possible to assume a functional link [64], as both the GABAergic and the glutamatergic systems have repeatedly been demonstrated to play a critical role in cognitive control processing [69,70]. Since cognitive control processes examined in the Simon task are modulated by the GABAergic system [69,70], it can be hypothesized that behavioral performance changes in the Simon Nogo task manifest when drinking lead to functionally relevant allostatic adaptations within the GABAergic transmitter system. However, studies employing other methods (such as PET) will be required to investigate and possibly substantiate such claims. In further support of the allostatic hypothesis, recent evidence suggests that allostatic effects induced by repeated drinking are characterized by a functional de-differentiation of neural networks. Specifically, repeated alcohol consumption is associated with a diffuse increase in neural activity across several neural networks, which ultimately decreases the specificity of information processing by the systems [71]. Based on the obtained results, one could argue that such a pattern of de-differentiation is also observed in the current behavioral and neurophysiological data, and becomes more pronounced with increasing drinking frequency.

The neurophysiological data further suggest that this functional allostasis does not affect all cognitive control processes equally, but is rather limited to very specific subprocesses involved in inhibitory control in the C-cluster. This cluster extracts the intermediate processes occurring between stimulus processing and response execution, and is thus thought to reflect functional operations such as response selection and decision making [48,72,73,74,75,76]. Only the centro-parietal P3 in the C-cluster revealed a decreased Simon Nogo effect (i.e., smaller differences between compatible and incompatible Nogo trials) in AUD participants, as compared with the control sample. Importantly, this lends further support to the dual-process account, which identified stimulus-response translation processes as a major factor driving effects in ‘Simon-like’ paradigms [77,78]. Source localization revealed that the smaller C-cluster Simon Nogo effect was associated with smaller activity modulations in the right inferior frontal gyrus (BA45 and BA9) and the middle frontal gyrus (BA46). Taken together, this provided neurophysiological evidence of a lower differentiation between congruent and incongruent Nogo trials in AUD participants, thereby matching the pattern of the behavioral results. The right inferior frontal gyrus is part of a cortical response inhibition network, which is activated whenever a motor response has to be inhibited [79,80,81,82,83,84]. This network has been associated with a breaking function needed to inhibit response-related processes reflected by the C-cluster [45], including in the Simon Nogo paradigm [85]. Activity differences between the groups in the Simon Nogo effect were also seen in the cuneus (BA17 and BA18) and precuneus (BA7), but the direction of effects was different (i.e., activity modulations were more pronounced in the AUD group). This suggests that the de-differentiation of processes may be specific for (pre)frontal brain regions and networks. Spatial attentional selection processes associated with parieto-occipital regions have frequently been shown to play a role in the Simon task [86,87,88,89,90]. However, these effects were not likely to have selectively altered direct route processing (i.e., selective attention to the task-irrelevant stimulus location), as this would have resulted in a larger (rather than a smaller) behavioral Simon effect. It could be speculated whether more differentiated processing in posterior brain regions might be linked to the less differentiated processing in prefrontal regions. While EEG connectivity analyses might provide some additional insights into this hypothesis, it would most likely be less spatially accurate than fMRI studies specifically designed to substantiate such claims.

Lastly, the MVPA analysis conducted across groups showed significant classification performance for stimulus-related processes (i.e., in the S-cluster) and central processes (i.e., in the C-cluster), with time windows corresponding well to those of the ERPs discussed above. This validates the importance of processes occurring within this time window to explain differential modulation of automated and controlled processes during inhibitory control. In the S-cluster, source localization analyses based on the MVPA-identified time windows showed differential activation by congruent vs. incongruent Nogo trials in the middle frontal gyrus (BA8 and BA6), precentral gyrus (BA9), cingulate gyrus (BA32), and middle and inferior temporal gyrus (BA37). In the C-cluster, differential activation was found in the post-central gyrus (BA1 and BA3) and primary motor cortex (BA4) (cf. Figure 5). The shared overlap with the regions found in the ERP group differences, especially in BA9, strongly suggests that the functional role of the rIFG in response inhibition may be one of the core factors that is altered in AUD, thereby driving the observed behavioral changes.

Although we can explain the obtained findings with the allostatic model of drinking states, it is important to note that AUD participants and healthy controls did not differ in their Simon effect in Go trials. This limits the role of allostatic processes in cognitive control functions in AUD, suggesting that allostatic effects may only be evident in case of specific (i.e., inhibitory) functions. In addition, the cause for the overall better inhibition response in AUD remains somewhat unclear, and we suggest assessing potential intrinsic motivation differences in future studies to rule out the possibility that participants in the AUD group may simply be more motivated to perform well when knowing about the investigated research question. Despite this, these findings provide an interesting insight on the relationship between AUD and inhibitory control deficits in addiction research. In particular, they provide evidence that the relationship between alcohol consumption and inhibition deficits may not be linear and only become generalized and/or evident in more severe AUD.

## 5. Conclusions

In summary, we showed that, compared with the control group, mild-to-moderate AUD participants showed overall better response inhibition performance. Furthermore, the AUD group was less influenced by the modulatory effect of automatic vs. controlled processes during response inhibition (i.e., Simon Nogo effect). Interestingly, the alcohol-related index of drinking frequency, instead of the proposed group distinction based on AUD criteria, predicted the level of the Simon Nogo effect. The neurophysiological data revealed that stimulus-response selection processes associated with inferior frontal and middle frontal gyrus regions especially are modulated differently in AUD participants. We suggest that the allostatic model of drinking effects can explain these results.

## Figures and Tables

**Table 1:** Sociodemographic measures for AUD and control participants.

	AUD		Control			
	*M (SEM)*	*Range*	*M (SEM)*	*Range*	*z*	*p*
**Age (years)**	27.07 (0.8)	18–40	27.06 (0.7)	18–40	−0.257	0.798
**Years of education**	12.70 (0.1)	9–13	12.16 (0.2)	9–13	−2.595	0.009 *
**BDI**	6.59 (0.7)	0–19	4.38 (0.5)	0–17	−2.216	0.027 *
**MMSE**	29.14 (0.1)	26–30	29.32 (0.1)	27–30	−1.258	0.208

Values are reported as the mean and standard error of the mean. Mann–Whitney U tests were used for statistical comparisons between the AUD and control groups. * denotes a significant effect at *p* < 0.05.

**Table 2:** Alcohol consumption measures for AUD and control participants.

	AUD		Control			
	*M (SEM)*	*Range*	*M (SEM)*	*Range*	*z*	*p*
**1-year AUD criteria**	4.41 (0.22)	2–9	0.25 (0.05)	0–1	−9.894	<0.001 *
**Lifetime AUD criteria**	4.17 (0.34)	0–10	0.66 (0.12)	0–4	−7.892	<0.001 *
**AUDIT score**	14.81 (0.70)	5–31	5.47 (0.39)	0–16	−8.608	<0.001 *
**Drinking frequency**	41.14 (2.52)	6–84	18.16 (1.85)	0–60	−6.270	<0.001 *
**Binge drinking frequency**	16.86 (1.89)	0–66	3.10 (0.57)	0–24	−6.734	<0.001 *

Values are reported as the mean and standard error of the mean. Mann–Whitney U tests were used for statistical comparisons between the AUD and control groups. The number of drinks was extrapolated from the amount and type of consumed alcoholic beverages. (Binge) drinking frequency is defined as the number of (binge) drinking events in the three months prior to the study. Detailed information on the conversion into standard drinks and more detailed data for 1-year and lifetime AUD criteria are reported in Tables S1 and S2 in the supplementary material, respectively. * denotes a significant effect at *p* < 0.05.

**Table 3:** Repeated-measure ANOVA results for main effects and interactions.

**Main Effects**	***F***	***p***	***η*** **^2^** ***_p_***
Group	8.120	0.005 *	0.063
Condition	24.856	<0.001 *	0.170
Congruency	10.533	0.002 *	0.080
**Interaction Effects**			
Condition × Congruency	42.296	<0.001 *	0.259
Group × Condition	14.388	<0.001*	0.106
Group × Congruency	1.916	0.169	0.016
Group × Condition × Congruency	4.414	0.038 *	0.035

* indicates values with *p* ≤ 0.05.

**Table 4:** ANCOVA results for main effects and interactions.

ANCOVA with AUD 1 Year			
*Effect*	*F*	*p*	*η* ^2^ *_p_*
Group	3.116	0.080	0.025
Condition	7.819	0.006 *	0.061
Congruency	0.233	0.630	0.002
AUD 1 year	0.171	0.680	0.001
***Interactions***			
Condition × Congruency	11.344	0.001 *	0.086
Group × Condition	1.809	0.181	0.015
Group × Congruency	2.726	0.101	0.022
Group × Condition × Congruency	0.383	0.537	0.003
Condition × AUD 1 year	0.370	0.544	0.003
Congruency × AUD 1 year	1.234	0.269	0.010
Condition × Congruency × AUD 1 year	0.232	0.631	0.002
**ANCOVA with AUDIT**			
***Effect***	***F***	***p***	***η* ^2^ *_p_***
Group	2.461	0.119	0.020
Condition	7.867	0.006 *	0.062
Congruency	1.970	0.163	0.016
AUDIT	0.229	0.633	0.002
***Interactions***			
Condition × Congruency	9.500	0.003 *	0.073
Group × Condition	3.508	0.064	0.028
Group × Congruency	0.667	0.416	0.006
Group × Condition × Congruency	0.946	0.333	0.008
Condition × AUDIT	0.880	0.350	0.007
Congruency × AUDIT	0.025	0.876	<0.001
Condition × Congruency × AUDIT	0.364	0.547	0.003
**ANCOVA with Drinking frequency**			
***Effect***	***F***	***p***	***η* ^2^ *_p_***
Group	1.046	0.308	0.009
Condition	17.127	<0.001 *	0.125
Congruency	21.392	<0.001 *	0.151
Drinking frequency	6.145	0.015 *	0.049
***Interactions***			
Condition × Congruency	18.645	<0.001 *	0.134
Group × Condition	4.503	0.036 *	0.036
Group × Congruency	0.495	0.483	0.004
Group × Condition × Congruency	1.103	0.296	0.009
Condition × Drinking frequency	3.541	0.062	0.029
Congruency × Drinking frequency	11.502	<0.001 *	0.087
Condition × Congruency × Drinking frequency	1.537	0.217	0.013
**ANCOVA with Binge drinking frequency ^1^**			
***Effect***	***F***	***p***	***η* ^2^ *_p_***
Group	5.772	0.018 *	0.046
Condition	13.267	<0.001 *	0.100
Congruency	5.514	0.021 *	0.044
Binge drinking	<0.001	0.984	<0.001
***Interactions***			
Condition × Congruency	31.653	<0.001 *	0.210
Group × Condition	9.686	0.002 *	0.075
Group × Congruency	1.030	0.312	0.009
Group × Condition × Congruency	1.031	0.312	0.009
Condition × Binge drinking	0.006	0.941	<0.001
Congruency × Binge drinking	0.012	0.914	<0.001
Condition × Congruency × Binge drinking	1.808	0.181	0.015

* indicates values with *p* ≤ 0.05. (Binge) drinking frequency is defined as the number of (binge) drinking events in the three months prior to the study. ^1^ For one subject in the control group, the response value to the binge drinking question was not recorded. Therefore, for this ANCOVA, the total subject sample was 122 instead of 123.

**Table 5:** Results of the multiple linear regression.

	*B*	*SE B*	*β*	*t*	*p*
Constant	−2.764	0.539		−5.129	<0.001 *
Group	0.468	1.078	0.083	0.434	0.665
AUD 1 year	−0.089	0.243	−0.075	−0.366	0.715
AUDIT	0.008	0.075	0.018	0.105	0.917
Drinking frequency	0.039	0.016	0.285	2.395	0.018 *
Binge drinking frequency	−0.001	0.031	−0.006	−0.045	0.964

Note. *F* = 2.346, *p* = 0.045, *R* = 0.303, *R*^2^ = 0.092; *B* = unstandardized regression coefficient; *SE B* = standard error of the coefficient; *β* = standard coefficient. (Binge) drinking frequency is defined as the number of (binge) drinking events in the three months prior to the study. * indicates values with *p* ≤ 0.05

## Data Availability

The data for the statistical analyses presented in this study are openly available in the Open Science Framework at DOI 10.17605/OSF.IO/8RC6D, or https://osf.io/8rc6d/. Personal requests for further information can be sent to all authors.

## Supplementary Materials

The following supporting information can be downloaded at: https://www.mdpi.com/article/10.3390/jcm11216557/s1, Drug and metabolites list; Table S1: Drinking frequency conversion table from units of alcohol in liters (l) to number of drinks; Figure S1: Standard ERPs and corresponding scalp topography maps in the S-cluster for P2 and N2 for Go and Nogo conditions; Figure S2: Standard ERPs and corresponding scalp topography maps in the C-cluster for Go trials and Nogo conditions for fronto-central P3 and centro-parietal P3 ERP.

## References

[1] Schuckit M.A. (2009). Alcohol-Use Disorders. Lancet.

[2] World Health Organisation (2019). Status Report on Alcohol Consumption Harm Policy Responses 30 European Countries 2019.

[3] Kranzler H.R., Soyka M. (2018). Diagnosis and Pharmacotherapy of Alcohol Use Disorder: A Review. JAMA.

[4] Liang J., Olsen R.W. (2014). Alcohol Use Disorders and Current Pharmacological Therapies: The Role of GABAA Receptors. Acta Pharmacol. Sin..

[5] Lingford-Hughes A., Welch S., Peters L., Nutt D. (2012). BAP Updated Guidelines: Evidence-Based Guidelines for the Pharmacological Management of Substance Abuse, Harmful Use, Addiction and Comorbidity: Recommendations from BAP. J. Psychopharmacol..

[6] Garbusow M., Sebold M., Beck A., Heinz A. (2014). Too Difficult to Stop: Mechanisms Facilitating Relapse in Alcohol Dependence. Neuropsychobiology.

[7] Grant B.F., Goldstein R.B., Saha T.D., Chou S.P., Jung J., Zhang H., Pickering R.P., Ruan W.J., Smith S.M., Huang B. (2015). Epidemiology of DSM-5 Alcohol Use Disorder. JAMA Psychiatry.

[8] Heinz A., Kiefer F., Smolka M.N., Endrass T., Beste C., Beck A., Liu S., Genauck A., Romund L., Banaschewski T. (2020). Addiction Research Consortium: Losing and Regaining Control over Drug Intake (ReCoDe)—From Trajectories to Mechanisms and Interventions. Addict. Biol..

[9] Stock A.-K. (2017). Barking up the Wrong Tree: Why and How We May Need to Revise Alcohol Addiction Therapy. Front. Psychol..

[10] Ghin F., Beste C., Stock A.-K. (2022). Neurobiological Mechanisms of Control in Alcohol Use Disorder—Moving towards Mechanism-Based Non-Invasive Brain Stimulation Treatments. Neurosci. Biobehav. Rev..

[11] Burchi E., Makris N., Lee M.R., Pallanti S., Hollander E. (2019). Compulsivity in Alcohol Use Disorder and Obsessive Compulsive Disorder: Implications for Neuromodulation. Front. Behav. Neurosci..

[12] Wilcox C.E., Dekonenko C.J., Mayer A.R., Bogenschutz M.P., Turner J.A. (2014). Cognitive Control in Alcohol Use Disorder: Deficits and Clinical Relevance. Rev. Neurosci..

[13] Sherman J.W., Gawronski B., Gonsalkorale K., Hugenberg K., Allen T.J., Groom C.J. (2008). The Self-Regulation of Automatic Associations and Behavioral Impulses. Psychol. Rev..

[14] Ulrich R., Schröter H., Leuthold H., Birngruber T. (2015). Automatic and Controlled Stimulus Processing in Conflict Tasks: Superimposed Diffusion Processes and Delta Functions. Cogn. Psychol..

[15] Botvinick M.M., Braver T.S., Barch D.M., Carter C.S., Cohen J.D. (2001). Conflict Monitoring and Cognitive Control. Psychol. Rev..

[16] Ridderinkhof R.K. (2002). Micro- and Macro-Adjustments of Task Set: Activation and Suppression in Conflict Tasks. Psychol. Res..

[17] Beste C., Moll C.K.E., Pötter-Nerger M., Münchau A. (2018). Striatal Microstructure and Its Relevance for Cognitive Control. Trends Cogn. Sci..

[18] Goschke T. (2003). Voluntary Action and Cognitive Control from a Cognitive Neuroscience Perspective. Voluntary Action: Brains, Minds, and Sociality.

[19] Goschke T. (2014). Dysfunctions of Decision-Making and Cognitive Control as Transdiagnostic Mechanisms of Mental Disorders: Advances, Gaps, and Needs in Current Research. Int. J. Methods Psychiatr. Res..

[20] Hommel B., Elliot A.J. (2015). Between Persistence and Flexibility: The Yin and Yang of Action Control. Advances in Motivation Science.

[21] Chmielewski W.X., Mückschel M., Beste C. (2018). Response Selection Codes in Neurophysiological Data Predict Conjoint Effects of Controlled and Automatic Processes during Response Inhibition. Hum. Brain Mapp..

[22] Opitz A., Hubert J., Beste C., Stock A.-K. (2019). Alcohol Hangover Slightly Impairs Response Selection but Not Response Inhibition. J. Clin. Med..

[23] Wendiggensen P., Ghin F., Koyun A.H., Stock A.-K., Beste C. (2022). Pretrial Theta Band Activity Affects Context-Dependent Modulation of Response Inhibition. J. Cogn. Neurosci..

[24] De Jong R., Liang C.-C., Lauber E. (1994). Conditional and Unconditional Automaticity: A Dual-Process Model of Effects of Spatial Stimulus-Response Correspondence. J. Exp. Psychol. Hum. Percept. Perform..

[25] Chmielewski W.X., Beste C. (2017). Testing Interactive Effects of Automatic and Conflict Control Processes during Response Inhibition—A System Neurophysiological Study. NeuroImage.

[26] Ghin F., Stock A.-K., Beste C. (2022). The Importance of Resource Allocation for the Interplay between Automatic and Cognitive Control in Response Inhibition—An EEG Source Localization Study. Cortex.

[27] Huster R.J., Enriquez-Geppert S., Lavallee C.F., Falkenstein M., Herrmann C.S. (2013). Electroencephalography of Response Inhibition Tasks: Functional Networks and Cognitive Contributions. Int. J. Psychophysiol..

[28] Eggert E., Takacs A., Münchau A., Beste C. (2022). On the Role of Memory Representations in Action Control: Neurophysiological Decoding Reveals the Reactivation of Integrated Stimulus–Response Feature Representations. J. Cogn. Neurosci..

[29] Petruo V., Takacs A., Mückschel M., Hommel B., Beste C. (2021). Multi-Level Decoding of Task Sets in Neurophysiological Data during Cognitive Flexibility. iScience.

[30] Prochnow A., Bluschke A., Weissbach A., Münchau A., Roessner V., Mückschel M., Beste C. (2021). Neural Dynamics of Stimulus-Response Representations during Inhibitory Control. J. Neurophysiol..

[31] Takacs A., Mückschel M., Roessner V., Beste C. (2020). Decoding Stimulus–Response Representations and Their Stability Using EEG-Based Multivariate Pattern Analysis. Cereb. Cortex Commun..

[32] Fahrenfort J.J., van Driel J., van Gaal S., Olivers C.N.L. (2018). From ERPs to MVPA Using the Amsterdam Decoding and Modeling Toolbox (ADAM). Front. Neurosci..

[33] King J.-R., Dehaene S. (2014). Characterizing the Dynamics of Mental Representations: The Temporal Generalization Method. Trends Cogn. Sci..

[34] Takács Á., Yu S., Mückschel M., Beste C. (2022). Protocol to Decode Representations from EEG Data with Intermixed Signals Using Temporal Signal Decomposition and Multivariate Pattern-Analysis. STAR Protoc..

[35] Treder M.S. (2020). MVPA-Light: A Classification and Regression Toolbox for Multi-Dimensional Data. Front. Neurosci..

[36] Bridwell D.A., Cavanagh J.F., Collins A.G.E., Nunez M.D., Srinivasan R., Stober S., Calhoun V.D. (2018). Moving Beyond ERP Components: A Selective Review of Approaches to Integrate EEG and Behavior. Front. Hum. Neurosci..

[37] Stock A.-K., Gohil K., Huster R.J., Beste C. (2017). On the Effects of Multimodal Information Integration in Multitasking. Sci. Rep..

[38] Yu S., Ghin F., Mückschel M., Ziemssen T., Stock A.-K., Beste C. (2022). A Role of the Norepinephrine System or Effort in the Interplay of Different Facets of Inhibitory Control. Neuropsychologia.

[39] Ouyang G., Herzmann G., Zhou C., Sommer W. (2011). Residue Iteration Decomposition (RIDE): A New Method to Separate ERP Components on the Basis of Latency Variability in Single Trials. Psychophysiology.

[40] Ouyang G., Sommer W., Zhou C. (2015). A Toolbox for Residue Iteration Decomposition (RIDE)—A Method for the Decomposition, Reconstruction, and Single Trial Analysis of Event Related Potentials. J. Neurosci. Methods.

[41] American Psychiatric Association (APA) (2013). Diagnostic and Statistical Manual of Mental Disorders: DSM-5^TM^.

[42] Babor T.F., Higgins-Biddle J.C., Saunders J., Monteiro M.G., World Health Organization (2001). AUDIT: The Alcohol Use Disorders Identification Test: Guidelines for Use in Primary Health Care.

[43] Chmielewski W.X., Zink N., Chmielewski K.Y., Beste C., Stock A.-K. (2020). How High-Dose Alcohol Intoxication Affects the Interplay of Automatic and Controlled Processes. Addict. Biol..

[44] Nunez P.L., Pilgreen K.L. (1991). The Spline-Laplacian in Clinical Neurophysiology: A Method to Improve EEG Spatial Resolution. J. Clin. Neurophysiol..

[45] Mückschel M., Dippel G., Beste C. (2017). Distinguishing Stimulus and Response Codes in Theta Oscillations in Prefrontal Areas during Inhibitory Control of Automated Responses. Hum. Brain Mapp..

[46] Opitz A., Beste C., Stock A.-K. (2020). Using Temporal EEG Signal Decomposition to Identify Specific Neurophysiological Correlates of Distractor-Response Bindings Proposed by the Theory of Event Coding. NeuroImage.

[47] Takács Á., Kóbor A., Kardos Z., Janacsek K., Horváth K., Beste C., Nemeth D. (2021). Neurophysiological and Functional Neuroanatomical Coding of Statistical and Deterministic Rule Information during Sequence Learning. Hum. Brain Mapp..

[48] Ouyang G., Hildebrandt A., Sommer W., Zhou C. (2017). Exploiting the Intra-Subject Latency Variability from Single-Trial Event-Related Potentials in the P3 Time Range: A Review and Comparative Evaluation of Methods. Neurosci. Biobehav. Rev..

[49] Ouyang G., Schacht A., Zhou C., Sommer W. (2013). Overcoming Limitations of the ERP Method with Residue Iteration Decomposition (RIDE): A Demonstration in Go/No-Go Experiments. Psychophysiology.

[50] Bradley A.P. (1997). The Use of the Area under the ROC Curve in the Evaluation of Machine Learning Algorithms. Pattern Recognit..

[51] Mazziotta J., Toga A., Evans A., Fox P., Lancaster J., Zilles K., Woods R., Paus T., Simpson G., Pike B. (2001). A Probabilistic Atlas and Reference System for the Human Brain: International Consortium for Brain Mapping (ICBM). Philos. Trans. R. Soc. Lond. B Biol. Sci..

[52] Fuchs M., Kastner J., Wagner M., Hawes S., Ebersole J.S. (2002). A Standardized Boundary Element Method Volume Conductor Model. Clin. Neurophysiol..

[53] Pascual-Marqui R.D. (2002). Standardized Low-Resolution Brain Electromagnetic Tomography (SLORETA): Technical Details. Methods Find. Exp. Clin. Pharmacol..

[54] Sekihara K., Sahani M., Nagarajan S.S. (2005). Localization Bias and Spatial Resolution of Adaptive and Non-Adaptive Spatial Filters for MEG Source Reconstruction. NeuroImage.

[55] Liu Y., van den Wildenberg W.P.M., de Graaf Y., Ames S.L., Baldacchino A., Bø R., Cadaveira F., Campanella S., Christiansen P., Claus E.D. (2019). Is (Poly-) Substance Use Associated with Impaired Inhibitory Control? A Mega-Analysis Controlling for Confounders. Neurosci. Biobehav. Rev..

[56] Kamarajan C., Porjesz B., Jones K.A., Choi K., Chorlian D.B., Padmanabhapillai A., Rangaswamy M., Stimus A.T., Begleiter H. (2005). Alcoholism Is a Disinhibitory Disorder: Neurophysiological Evidence from a Go/No-Go Task. Biol. Psychol..

[57] Corbit L.H., Janak P.H. (2016). Habitual Alcohol Seeking: Neural Bases and Possible Relations to Alcohol Use Disorders. Alcohol. Clin. Exp. Res..

[58] Garbusow M., Schad D.J., Sommer C., Jünger E., Sebold M., Friedel E., Wendt J., Kathmann N., Schlagenhauf F., Zimmermann U.S. (2014). Pavlovian-to-Instrumental Transfer in Alcohol Dependence: A Pilot Study. Neuropsychobiology.

[59] Sommer C., Garbusow M., Jünger E., Pooseh S., Bernhardt N., Birkenstock J., Schad D.J., Jabs B., Glöckler T., Huys Q.M. (2017). Strong Seduction: Impulsivity and the Impact of Contextual Cues on Instrumental Behavior in Alcohol Dependence. Transl. Psychiatry.

[60] Campbell J., Sharma A. (2013). Compensatory Changes in Cortical Resource Allocation in Adults with Hearing Loss. Front. Syst. Neurosci..

[61] Geisler M.W., Murphy C. (2000). Event-Related Brain Potentials to Attended and Ignored Olfactory and Trigeminal Stimuli. Int. J. Psychophysiol..

[62] Sugimoto F., Katayama J. (2013). Somatosensory P2 Reflects Resource Allocation in a Game Task: Assessment with an Irrelevant Probe Technique Using Electrical Probe Stimuli to Shoulders. Int. J. Psychophysiol..

[63] Kubo-Kawai N., Kawai N. (2010). Elimination of the Enhanced Simon Effect for Older Adults in a Three-Choice Situation: Ageing and the Simon Effect in a Go/No-Go Simon Task. Q. J. Exp. Psychol..

[64] Correas A., Cuesta P., Rosen B.Q., Maestu F., Marinkovic K. (2021). Compensatory Neuroadaptation to Binge Drinking: Human Evidence for Allostasis. Addict. Biol..

[65] Koob G., Le Moal M. (2001). Drug Addiction, Dysregulation of Reward, and Allostasis. Neuropsychopharmacology.

[66] Marinkovic K., Myers A.B.A., Arienzo D., Sereno M.I., Mason G.F. (2022). Cortical GABA Levels Are Reduced in Young Adult Binge Drinkers: Association with Recent Alcohol Consumption and Sex. NeuroImage Clin..

[67] McEwen B. (2000). Allostasis and Allostatic Load Implications for Neuropsychopharmacology. Neuropsychopharmacology.

[68] Roberto M., Varodayan F.P. (2017). Synaptic Targets: Chronic Alcohol Actions. Neuropharmacology.

[69] Haag L., Quetscher C., Dharmadhikari S., Dydak U., Schmidt-Wilcke T., Beste C. (2015). Interrelation of Resting State Functional Connectivity, Striatal GABA Levels, and Cognitive Control Processes. Hum. Brain Mapp..

[70] Dharmadhikari S., Ma R., Yeh C.-L., Stock A.-K., Snyder S., Zauber S.E., Dydak U., Beste C. (2015). Striatal and Thalamic GABA Level Concentrations Play Differential Roles for the Modulation of Response Selection Processes by Proprioceptive Information. Neuroimage.

[71] Pérez-Ramírez Ú., López-Madrona V.J., Pérez-Segura A., Pallarés V., Moreno A., Ciccocioppo R., Hyytiä P., Sommer W.H., Moratal D., Canals S. (2022). Brain Network Allostasis after Chronic Alcohol Drinking Is Characterized by Functional Dedifferentiation and Narrowing. J. Neurosci..

[72] Bluschke A., Chmielewski W.X., Mückschel M., Roessner V., Beste C. (2017). Neuronal Intra-Individual Variability Masks Response Selection Differences between ADHD Subtypes—A Need to Change Perspectives. Front. Hum. Neurosci..

[73] Mückschel M., Chmielewski W., Ziemssen T., Beste C. (2017). The Norepinephrine System Shows Information-Content Specific Properties during Cognitive Control—Evidence from EEG and Pupillary Responses. NeuroImage.

[74] Verleger R., Metzner M.F., Ouyang G., Śmigasiewicz K., Zhou C. (2014). Testing the Stimulus-to-Response Bridging Function of the Oddball-P3 by Delayed Response Signals and Residue Iteration Decomposition (RIDE). Neuroimage.

[75] Verleger R., Siller B., Ouyang G., Śmigasiewicz K. (2017). Effects on P3 of Spreading Targets and Response Prompts Apart. Biol. Psychol..

[76] Wolff N., Mückschel M., Beste C. (2017). Neural Mechanisms and Functional Neuroanatomical Networks during Memory and Cue-Based Task Switching as Revealed by Residue Iteration Decomposition (RIDE) Based Source Localization. Brain Struct. Funct..

[77] Hommel B. (2011). The Simon Effect as Tool and Heuristic. Acta Psychol..

[78] Keye D., Wilhelm O., Oberauer K., Stürmer B. (2013). Individual Differences in Response Conflict Adaptations. Front. Psychol.

[79] Aron A.R., Robbins T.W., Poldrack R.A. (2004). Inhibition and the Right Inferior Frontal Cortex. Trends Cogn. Sci..

[80] Aron A.R., Cai W., Badre D., Robbins T.W. (2015). Evidence Supports Specific Braking Function for Inferior PFC. Trends Cogn. Sci..

[81] Garavan H., Hester R., Murphy K., Fassbender C., Kelly C. (2006). Individual Differences in the Functional Neuroanatomy of Inhibitory Control. Brain Res..

[82] Kelly A.M.C., Hester R., Murphy K., Javitt D.C., Foxe J.J., Garavan H. (2004). Prefrontal-Subcortical Dissociations Underlying Inhibitory Control Revealed by Event-Related FMRI. Eur. J. Neurosci..

[83] Konishi S., Nakajima K., Uchida I., Sekihara K., Miyashita Y. (1998). No-Go Dominant Brain Activity in Human Inferior Prefrontal Cortex Revealed by Functional Magnetic Resonance Imaging. Eur. J. Neurosci..

[84] Bari A., Robbins T.W. (2013). Inhibition and Impulsivity: Behavioral and Neural Basis of Response Control. Prog. Neurobiol..

[85] Wolff N., Chmielewski W., Buse J., Roessner V., Beste C. (2019). Paradoxical Response Inhibition Advantages in Adolescent Obsessive Compulsive Disorder Result from the Interplay of Automatic and Controlled Processes. NeuroImage Clin..

[86] Abrahamse E.L., Van der Lubbe R.H.J. (2008). Endogenous Orienting Modulates the Simon Effect: Critical Factors in Experimental Design. Psychol. Res..

[87] Vahid A., Mückschel M., Stober S., Stock A.-K., Beste C. (2020). Applying Deep Learning to Single-Trial EEG Data Provides Evidence for Complementary Theories on Action Control. Commun. Biol..

[88] Wascher E., Schatz U., Kuder T., Verleger R. (2001). Validity and Boundary Conditions of Automatic Response Activation in the Simon Task. J. Exp. Psychol. Hum. Percept. Perform..

[89] Wiegand K., Wascher E. (2005). Dynamic Aspects of Stimulus-Response Correspondence: Evidence for Two Mechanisms Involved in the Simon Effect. J. Exp. Psychol. Hum. Percept. Perform..

[90] Bensmann W., Zink N., Werner A., Beste C., Stock A.-K. (2020). Acute Alcohol Effects on Response Inhibition Depend on Response Automatization, but Not on GABA or Glutamate Levels in the ACC and Striatum. J. Clin. Med..

