# Supplementary material for: On the Role of Stimulus-Response Context in Inhibitory Control in Alcohol Use Disorder
Source: J Clin Med. 2022 Nov 4;11(21):6557. doi: 10.3390/jcm11216557 (PMC9657501; doi:10.3390/jcm11216557)
Supplement: Supplementary file 1 [file jcm-11-06557-s001.zip › jcm-1999468-supplementary.pdf]

Journal of
Clinical Medicine

MDPI

Supplementary Materials

1. Drugs/metabolites participants were tested for

Using a rapid urine screening test (SureStep™, Innovacon Inc, USA), we tested for traces/metabolites of amphetamines, barbiturates, benzodiazepines, buprenorphine, clonazepam, cocaine, cotinine, fentanyl, heroin, ketamine, methamphetamine, morphine, opiate, oxycodone phencyclidine, propoxyphene, tramadol and tricyclic antidepressants.

2. Drinking frequency conversion table from alcoholic beverages in liters (l) to the number of standard drinks

Table S1. Number of standard drinks per serving size of different alcoholic beverages in liters (l).

Type of alcoholic beverage	Conversion of beverage quantity to standard drinks		
Beer	0.3l = 1.5 drinks	0.5 = 2.5 drinks	1l = 5 drinks
Wine	0.2l = 1.8 drinks	0.7 = 6.5 drinks	1.0l = 9 drinks
Liquor wine	0.05l = 0.75 drinks	0.1l =1.5 drinks	0.2l = 3 drinks
Sparkling wine	0.1l = 1 drink	0.2l = 2 drinks	0.75l = 8 drinks
Spirits & hard liquors	0.02l = 0.75 drinks	0.04l = 1.5 drinks	0.7l = 25 drinks
Sweet liquors	0.02 = 0.5 drinks	0.04l = 2 drinks	0.08l = 4 drinks
Cocktails	0.02l = 0.75 drinks	0.04l = 1.5 drinks	0.08l = 3 drinks

3. Detailed frequency table of 1-year and lifetime AUD criteria in each group

Table S2: Frequency table for AUD 1-year criteria and AUD lifetime criteria for AUD and control participants.

**1-year AUD criteria**	**AUD (n = 59)**		**Controls (n = 64)**	
	N	*Percentage*	*N*	*Percentage*
0	0	0	48	75%
1	0	0	16	25%
2	6	10.2%	0	0
3	12	20.3%	0	0
4	19	32.2%	0	0
5	8	13.6%	0	0
6	6	10.2%	0	0
7	5	8.5%	0	0
8	2	3.4%	0	0
9	1	1.7%	0	0
10	0	0	0	0
**Lifetime AUD criteria**	*N*	*Percentage*	*N*	*Percentage*
0	4	6.8%	39	60.9%
1	5	8.5%	13	20.3%
2	10	16.9%	8	12.5%
3	6	10.2%	3	4.7%
4	7	11.9%	1	1.6%
5	12	20.3%	0	0
6	3	5.1%	0	0
7	4	6.8%	0	0
8	5	8.5%	0	0
9	1	1.7%	0	0
10	2	3.4%	0	0

Note: All of the participants included in the control group (i.e., AUD 1-year criteria ≤1) who reported lifetime AUD criteria ≥ 2 were drinking within normal range for the past 3 months prior to the experiment (i.e., drinking frequency always ≥1).

4. Additional two-way mixed-effects ANCOVAs and multiple linear regression analyses investigating the effects of BDI and years of education onto behavioral performance (accuracy).[1]

Table S3: ANCOVA results for main effects and interactions.

**ANCOVA with BDI**			
Effect	***F***	***p***	η^2_p
Group	8.505	0.004*	0.066
Condition	11.645	<0.001*	0.088
Congruency	6.453	0.012*	0.051
BDI	0.424	0.516	0.004
Interactions			
Condition x Congruency	19.582	<0.001*	0.140
Group x Condition	13.246	<0.001*	0.099
Group x Congruency	1.496	0.224	0.012
Group x Condition x Congruency	3.970	0.049*	0.032
Condition x BDI	0.034	0.853	<0.001
Congruency x BDI	0.293	0.589	0.002
Condition x Congruency x BDI	0.043	0.837	<0.001
**ANCOVA with years of education**			
Effect	***F***	***p***	η^2_p
Group	7.934	0.006*	0.067
Condition	0.908	0.343	0.008
Congruency	0.352	0.554	0.003
Education	0.025	0.874	<0.001
Interactions			
Condition x Congruency	2.765	0.099	0.024
Group x Condition	13.019	<0.001*	0.105
Group x Congruency	1.119	0.292	0.010
Group x Condition x Congruency	2.906	0.091	0.026
Condition x Education	0.242	0.624	0.002
Congruency x Education	0.082	0.775	0.001
Condition x Congruency x Education	1.129	0.290	0.010

Note. Significant p values (<0.05) are marked with an asterisk.

[1] For n = 9 (AUD = 3, controls = 6) subjects , the years of eduction were not provided due to ambiguous questionnaire responses by the respective participants. Thus, ANCOVA and regression analyses with years of eduction were conducted on a sample of n = 114 (AUD = 58; controls = 56) participants.

Table S4. Results of the multiple linear regression

	B	*SE B*	*β*	*t*	*p*
Constant	-4.717	2.843		-1.659	0.100
Group	0.841	0.557	0.150	1.510	0.134
BDI	-0.028	0.056	0.049	0.505	0.614
Years of education	0.205	0.229	0.086	0.895	0.373

Note. F=1.573, p = *0.200*, R=0.203, R^2=0.041; B = unstandardized regression coefficient; $SE\ B$ = standard error of the coefficient; β = standard coefficient.

5.Supplementary figures for the S-cluster and the C-cluster

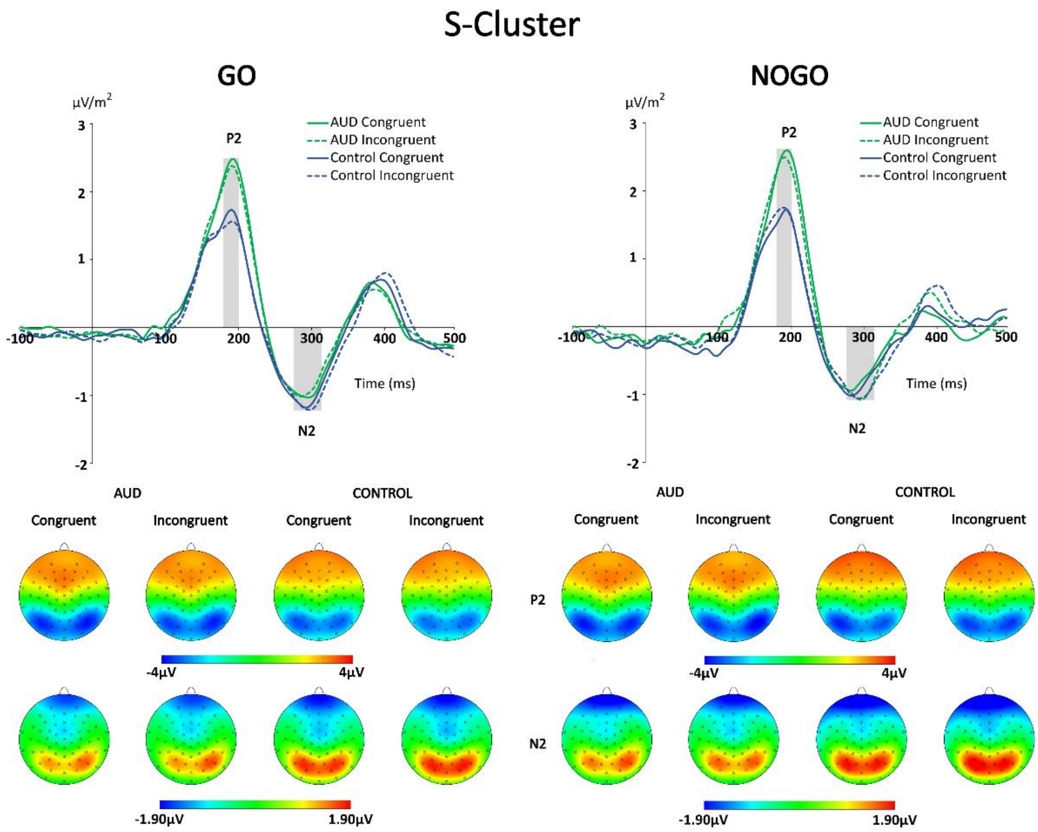

Figure S1. P2 and N2 ERPs and corresponding scalp topography maps in the S-cluster for Go trials (left panel) and Nogo trials (right panel). Both ERPs are pooled across electrodes FCz and Cz Blue lines denote the ERPs of the control group and green lines those of the AUD group. Solid lines indicate congruent trials and dashed lines indicate incongruent trials. The gray areas illustrate the time-windows used for the statistical analyses.

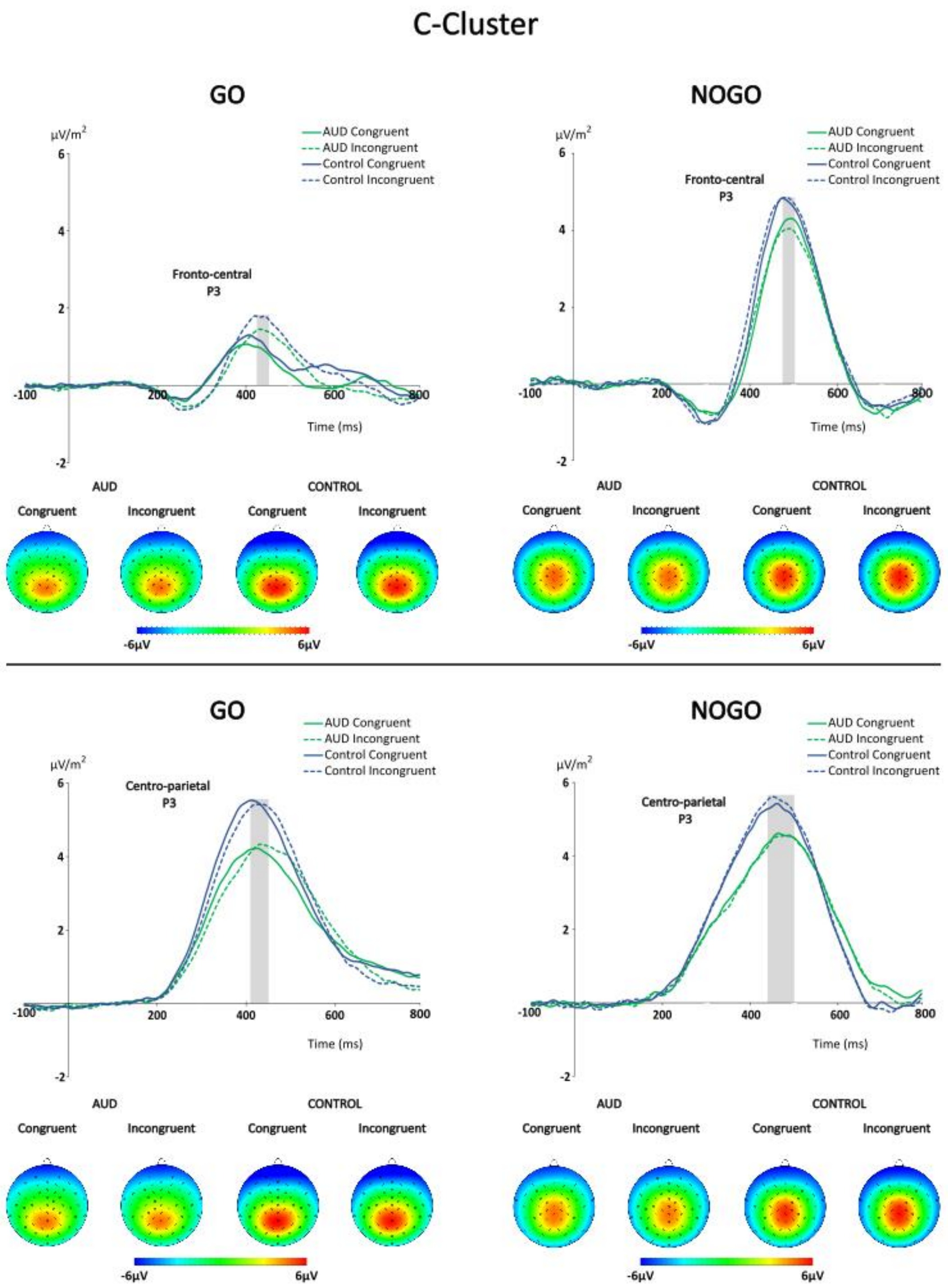

Figure S2. ERPs and corresponding scalp topography maps in the C-cluster for Go trials (left panel) and Nogo trials (right panel) for the fronto-central P3 pooled across electrodes FCz and Cz (top) and the centro-parietal P3 pooled across electrodes Cz and CPz (bottom). Blue lines denote the ERPs of the control group and green lines those of the AUD group. Solid lines indicate congruent trials and dashed lines indicate incongruent trials. The gray areas illustrate the time-windows used for the statistical analyses.
